# Supplementary material for: Thermal coupling of the Indo-Pacific warm pool and Southern Ocean over the past 30,000 years
Source: Nat Commun. 2022 Sep 17;13:5457. doi: 10.1038/s41467-022-33206-y (PMC9482618; doi:10.1038/s41467-022-33206-y)
Supplement: Supplementary file 1 — Supplementary Information [file 41467_2022_33206_MOESM1_ESM.pdf]

Supplementary Information for

Thermal coupling of the Indo-Pacific warm pool and Southern Ocean over the past 30,000 years

Shuai Zhang [1], Zhoufei Yu [2*], Yue Wang [3], Xun Gong [4, 5, 6], Ann Holbourn [7], Fengming Chang [8, 9], Heng Liu [1], Xuhua Cheng [1] and Tiegang Li [9, 10*]

[1] *College of Oceanography, Hohai University, Nanjing, 210098, China*;

[2] *State Key Laboratory of Palaeobiology and Stratigraphy, Nanjing Institute of Geology and Palaeontology, Chinese Academy of Sciences, Nanjing, 210008, China*;

[3] *State Key Laboratory of Marine Geology, Tongji University, Shanghai, 200092, China*;

[4] *Institute for Advanced Marine Research, China University of Geosciences, Guangzhou, 511455, China*;

[5] *State Key Laboratory of Biogeology and Environmental Geology, Hubei Key Laboratory of Marine Geological Resources, China University of Geosciences, Wuhan, 430074 China*;

[6] *Shandong Provincial Key Laboratory of Computer Networks, Qilu University of Technology (Shandong Academy of Sciences), Jinan, 250014, China*;

[7] *Institute of Geosciences, Christian-Albrechts-University, Kiel, D-24118, Germany*;

[8] *Key Laboratory of Marine Geology and Environment, Institute of Oceanology, Chinese Academy of Sciences, Qingdao, 266071, China*;

[9] *Laboratory for Marine Geology, Pilot National Laboratory for Marine Science and Technology (Qingdao), Qingdao, 266237, China*;

[10] *Key Laboratory of Marine Sedimentology and Environmental Geology, First Institute of Oceanography, MNR, Qingdao 266061, China*

This PDF file includes:

1. Statistical results: Supplementary Figures 1 to 11 and Table 1.
2. Seasonality of temperature records since 30 ka within the IPWP region.
3. Estimation of ocean heat content in core KX22-4.
   3.1. Age model of core KX22-4.
   3.2. Estimations of foraminiferal calcification depth in core KX22-4.
   3.3. Calculation of the upper ocean heat content (OHC) in core KX22-4.

Supplementary References.

* *Correspondence to:* zfyu@nigpas.ac.cn

* *Correspondence to:* tgli@fio.org.cn

1. Statistical results

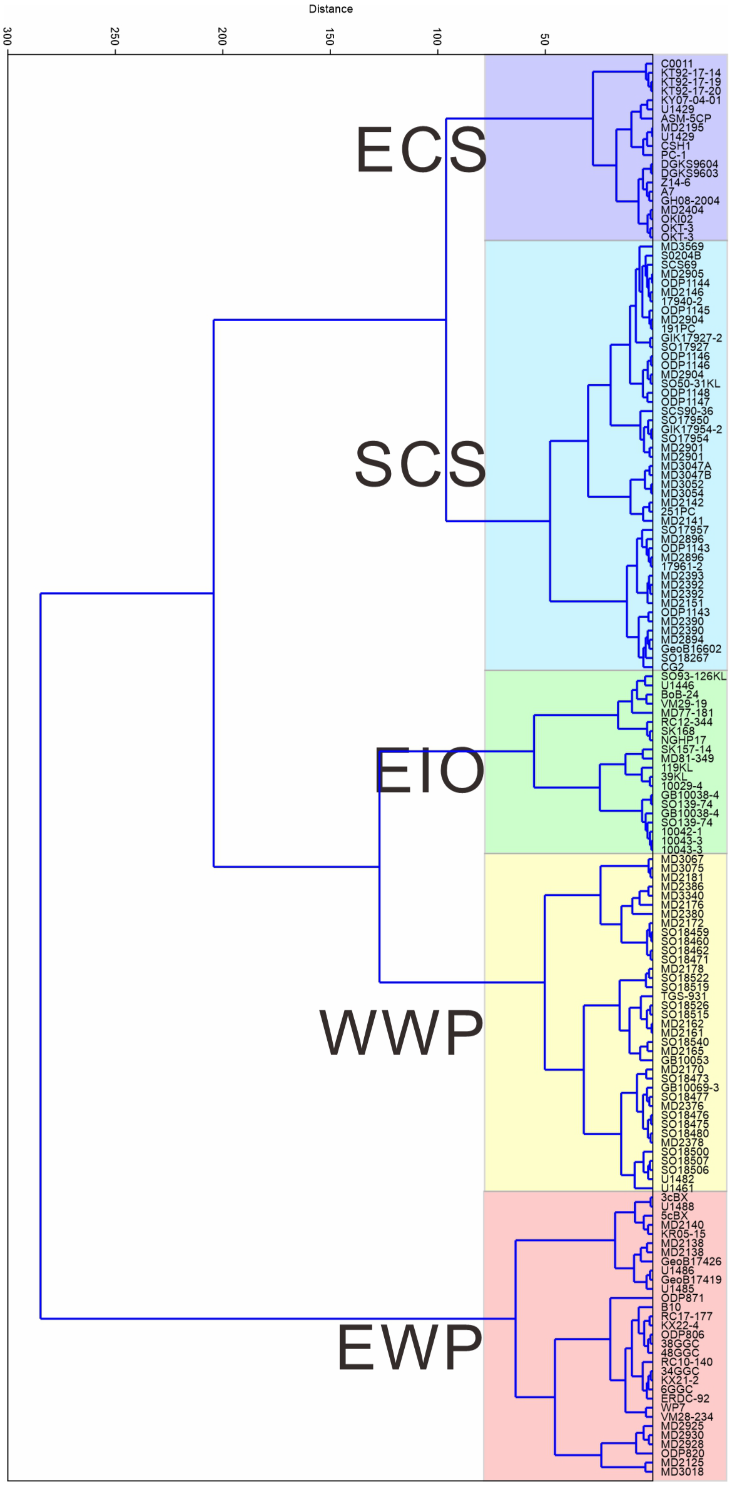

Supplementary Fig. 1. Result of cluster analysis of Indo-Pacific SST records. ECS: East China Sea; SCS: South China Sea; EIO: eastern Indian Ocean; WWP and EWP: western and eastern parts of the western Pacific warm pool.

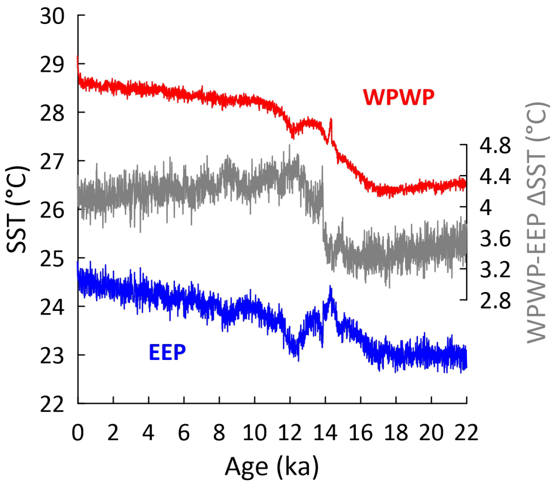

Supplementary Fig. 2. Transient simulation (TRACE) of SST variations in the WPWP and EEP regions during the last 22 kyr. Mean SSTs of the WPWP in the early Holocene (EH) and last glacial (LG) are 28.30±0.09°C and 26.45±0.08°C, respectively. Mean SSTs of the EEP in the EH and LG are 24.03±0.18°C and 23.01±0.15°C, respectively. Data are from https://www.earthsystemgrid.org/project/trace.html[1].

Supplementary Tab. 1. Mean SST over selected time intervals and the ΔSST between the LG and EH in the equatorial Pacific. WPWP, CEP and EEP represent the core region of the western Pacific warm pool, central equatorial Pacific and eastern equatorial Pacific, respectively. Modern SST are from the World Ocean Atlas 2018 dataset[2].

Sea Area	Modern SST (°C)	EH SST (°C)	LG SST (°C)	LG-EH ΔSST (°C)
**WPWP**	**29.10** ± 0.61	**28.74** ± 0.77[*]	**26.30** ± 0.86[*]	**-2.41** ± 0.63[*]
**CEP**	**27.58** ± 0.73	**27.03** ± 0.64[*]	**25.23** ± 0.70[*]	**-1.77** ± 0.45[*]
**EEP**	**23.69** ± 1.69	**23.76** ± 1.56[*#]	**22.47** ± 1.49[*#]	**-1.50** ± 0.60[*#]
	**23.60** ± 1.75	**23.69** ± 1.97[*]	**22.35** ± 1.65[*]	**-1.62** ± 0.75[*]
	**23.74** ± 1.65	**23.81** ± 1.20[#]	**22.54** ± 1.39[#]	**-1.43** ± 0.49[#]

[*]SST from Mg/Ca measurement; [#]SST from $U^{k'}_{37}$ measurement.

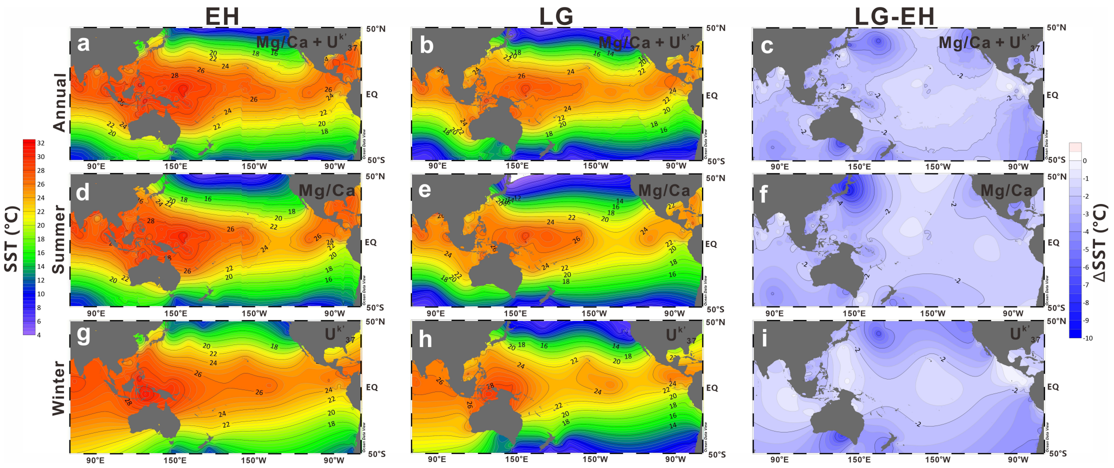

Supplementary Fig. 3. SST distributions based on published data in the Pacific during the EH (a, d, g) **and LG** (b, e, h)**, and the difference between these two periods** (c, f, i).

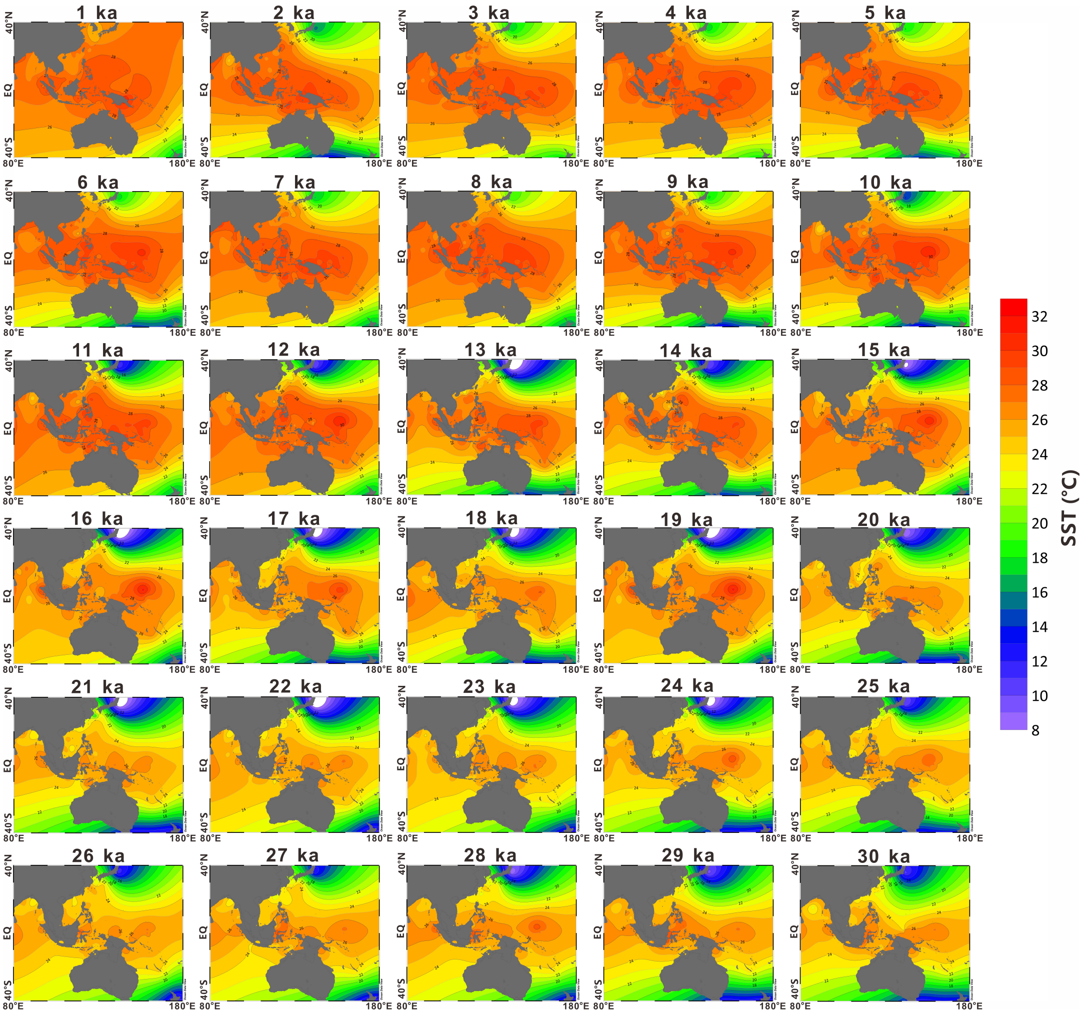

Supplementary Fig. 4. Variations of SST distribution based on published data in the IPWP during the last 30 kyr with 1 kyr time interval.

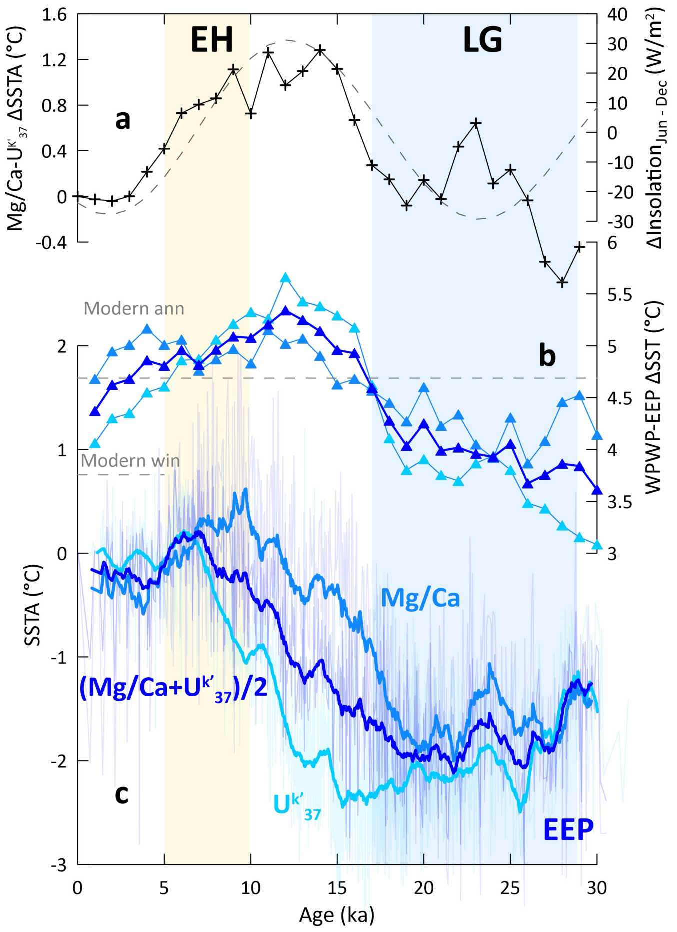

Supplementary Fig. 5. SSTA stacks in the EEP region during the last 30 kyr. (a) Comparison of SSTA differences between Mg/Ca and $U^{k'}_{37}$ data (black) with the insolation difference at the equator between June and December (gray dashed). (b) Zonal SST difference between the WPWP (Mg/Ca) and EEP (dodger blue for Mg/Ca, sky blue for $U^{k'}_{37}$ and blue for average of Mg/Ca and $U^{k'}_{37}$). (c) SSTA stacks from Mg/Ca (dodger blue), $U^{k'}_{37}$ (sky blue) and their average value (blue) in the EEP.

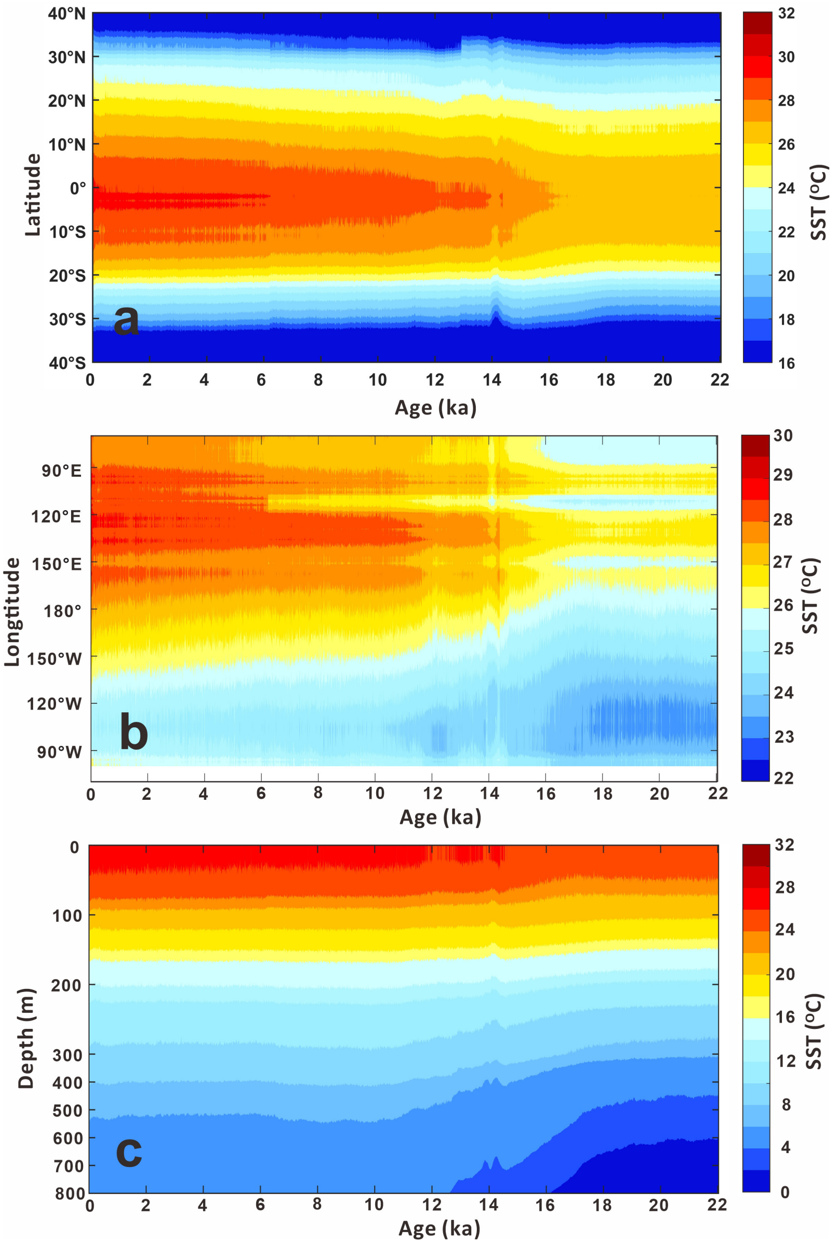

Supplementary Fig. 6. Transient simulation (TRACE) result of spatial SST evolution during the last 22 kyr. (a) Mean SST evolution within 110–130°E along 40°N–40°S transect. (b) Mean SST evolution within 10°N–10°S along 70°E–70°W transect. (c) Vertical temperature evolution across 0–800 m water depth at Site KX22-4. Data are from https://www.earthsystemgrid.org/project/trace.html[1].

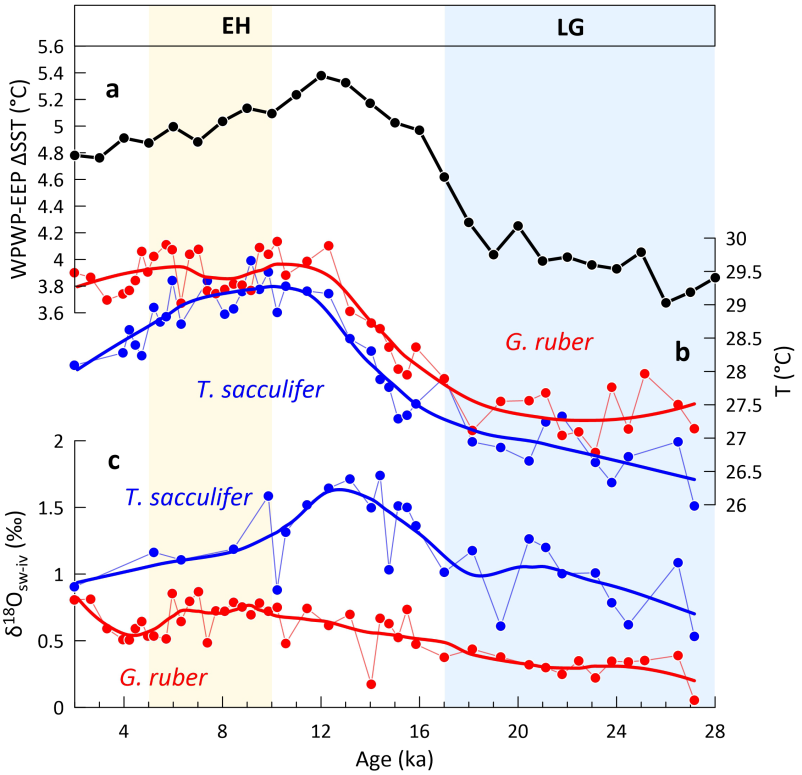

Supplementary Fig. 7. Variations of the upper water column at the location of core KX22-4 in the WPWP since 30 ka. (a) Zonal SST gradient between the WPWP and EEP. (b) Mg/Ca temperature reconstructed from *G. ruber*[3] and *T. sacculifer*. (c) Residual seawater $\delta^{18}O$ ($\delta^{18}O_{sw\text{-}iv}$) from these two foraminiferal species. Temperature of *T. sacculifer* was calculated with the equation Mg/Ca = 0.24×exp (0.097×T) from the western Pacific[4]. Seawater $\delta^{18}O$ ($\delta^{18}O_{sw}$) was calculated with the equation T = 16.5 – 4.8×($\delta^{18}O$ - $\delta^{18}O_{sw}$) with a 0.27‰ correction[5]. $\delta^{18}O_{sw\text{-}iv}$ was calculated with a correction for ice volume change[6].

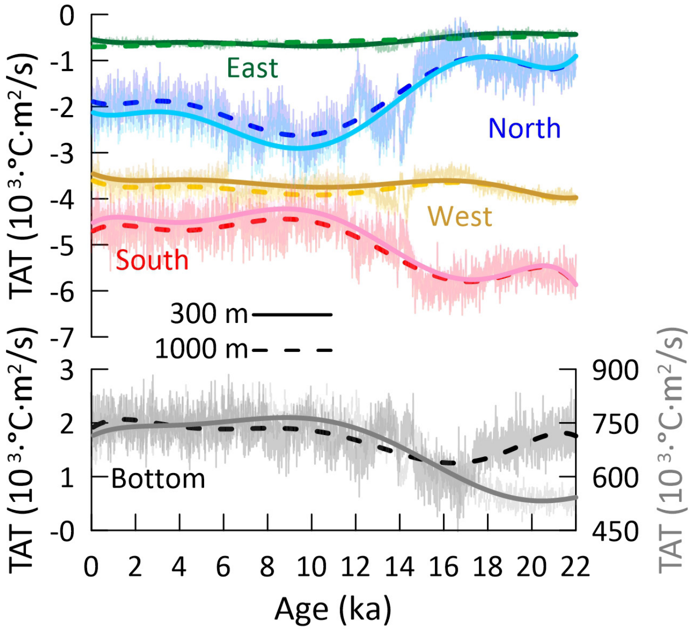

Supplementary Fig. 8. The horizontal (from the east, west, north and south) and vertical (from below) temperature advection tendency (TAT) toward the box (5°N-5°S, 120-170°E, 0-300/1000 m) from TRACE

result since 30 ka. Positive trends (from the south and below) represent heat absorption into the box, and negative trends represent heat release from the box (to the east, west and north) during the deglacial period. Data are from https://www.earthsystemgrid.org/project/trace.html[1].

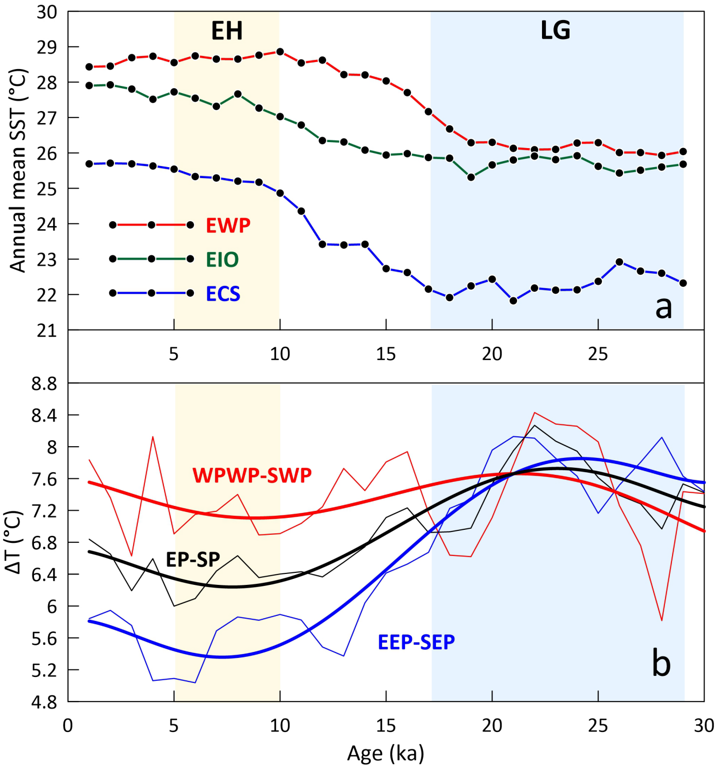

Supplementary Fig. 9. SST variations in the Pacific during the last 30 kyr. (a) Interpolated 1 kyr annual mean SSTs (average of Mg/Ca and $U^{k'}_{37}$) in the eastern part of the warm pool (EWP), eastern Indian Ocean (EIO) and East China Sea (ECS). (b) SST difference between the equatorial Pacific (EP) and south Pacific (SP). The red line represents the SST difference between the WPWP and southwestern Pacific (SWP), the blue line represents the SST difference between the EEP and southeastern Pacific (SEP). EP-SP represents the mean value of the differences. Bold lines are polynomial fitting results for interpolated 1 kyr SST difference between regions.

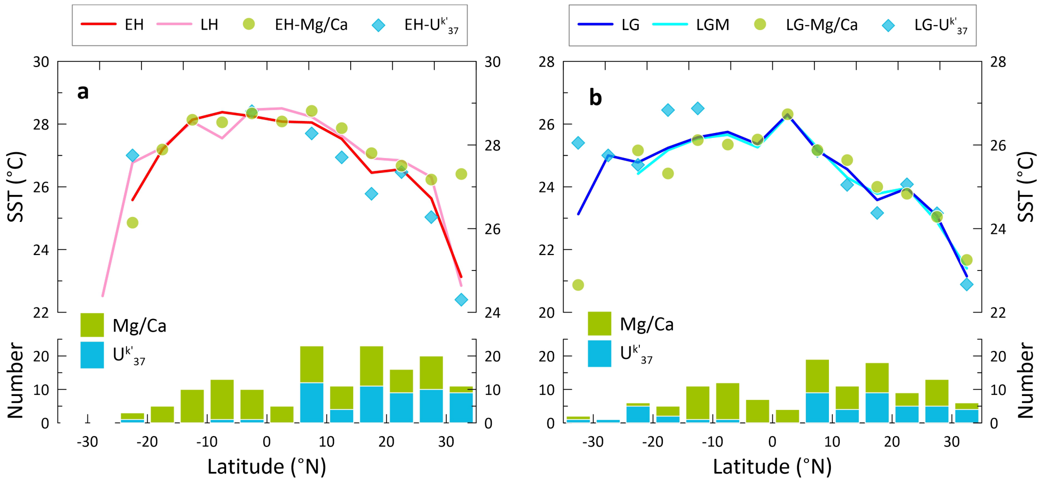

Supplementary Fig. 10. Meridional distributions of mean SST values and number of records in the Holocene (a) and Last Glacial (b) periods. EH, LH, LG and LGM represent the early Holocene (5–10 ka), late Holocene (0–

5 ka), last glacial period (17–29 ka) and last glacial maximum (19–23 ka), respectively.

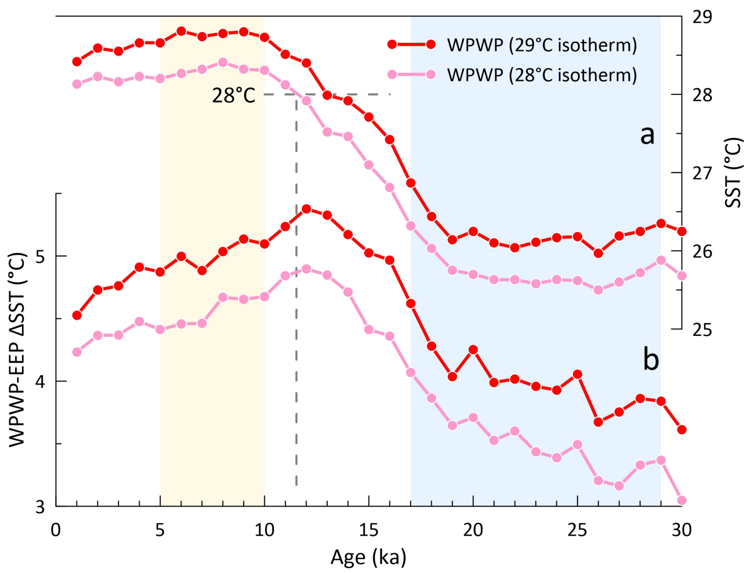

Supplementary Fig. 11. Comparison of results from sites within the 29°C and 28°C isotherms. (a) Mg/Ca SST stacks. (b) ΔSST between the WPWP and EEP. The dotted line marks the time when the 28°C warm pool reached its modern extent.

2. Seasonality of temperature records since 30 ka within the IPWP region

The published SST records within IPWP region were mainly derived from Mg/Ca and $U^{k'}_{37}$ measurements. Comparison between these records showed that $U^{k'}_{37}$-SST differs substantially from Mg/Ca-SST in most regions, except for the equatorial WPWP characterized by weak seasonality during the Holocene (Supplementary Fig. 12). Many studies have reported that foraminiferal Mg/Ca and $U^{k'}_{37}$ might reflect different seasonal SST signals, related to the specific ecology (habitat depth) and seasonality (production season) of the foraminifera and coccolithophores[7,8]. These studies suggested that alkenone reconstructed SST indicates tropical winter[9,10] or annual temperature[8,11] based on the phytoplankton blooming period, whereas Mg/Ca in *G. ruber* records summer[10-12] or annual SST[13,14]. Sediment trap data revealed that the peaks of *G. ruber* fluxes in the western equatorial Pacific[13], South China Sea[15] and northwest Pacific Ocean[16] occurred primarily in the summer and early fall. Furthermore, foraminiferal Mg/Ca and $U^{k'}_{37}$ based SST anomaly (SSTA) records from these four regions spanning the past 30 kyr (Supplementary Fig. 13a, c) revealed different patterns of variability mainly during the early Holocene (EH, 5–10 ka). Consistently high levels for Mg/Ca-SSTA in contrast to gradually rising trends for $U^{k'}_{37}$-SSTA exactly matched the

transient climate simulations of SSTA in Jun-Aug-Sep (JAS) and Dec-Jan-Feb (DJF)[10] (Supplementary Fig. 13b), respectively. Hence, the differences in Mg/Ca and $U^{k'}_{37}$ based temperature behaviors appear to be linked to the seasons, when various species are dominant.

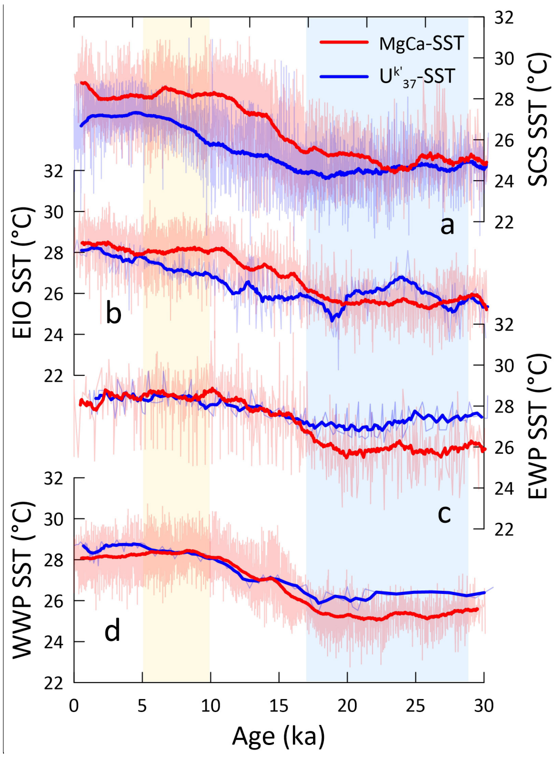

Supplementary Fig. 12. Mg/Ca and $U^{k'}_{37}$ derived SST stacks for the South China Sea (a), eastern Indian Ocean (b), eastern (c) and western (d) parts of the western Pacific warm pool within the IPWP region. Shadow curves show the raw data. SST stacks were computed using a sliding rectangular window of 1-kyr width by STATNARY 1.2 (https://www.marum.de/Prof.-Dr.-michael-schulz/Michael-Schulz-Software.html). Magenta and cyan bars indicate the Early Holocene and last glacial periods, respectively.

Moreover, the ΔSST between the last glacial period (LG, 17–29 ka) and EH based on $U^{k'}_{37}$ data is of smaller magnitude than Mg/Ca derived estimates (0.59–1.45°C lower, Tab. s2) and is comparable with the MARGO global SST reconstruction[10]. This may imply more intense warming in summer than in winter since the LG (Supplementary Fig. 12), which is consistent with a study of $\delta^{18}O$ composition of individual foraminifera from the western Arabian Sea[17]. For the South China Sea and Eastern Indian Ocean, the SST seasonality was enhanced in the deglaciation owing to more rapid and earlier summer warming (Supplementary Fig. 12 and Fig. 13), and it was stronger in the EH than in the LG (see the difference between Mg/Ca and $U^{k'}_{37}$ based data in Supplementary Fig. 12), which is consistent with one seasonality study on the Sunda Shelf[18]. These seasonal SST

responses may be induced by the higher seasonality in low-latitude insolation during the EH compared to the LG, which exhibited higher summer and lower winter solar radiation in the EH than in the LG (Supplementary Fig. 14), modulated by lower precession during the EH. On the other hand, the albedo feedback of a highly variable boreal snow and sea-ice cover would enhance winter cooling and summer warming in the EH, and the increased water vapor compared to the LG would also enhance summer warming owing to the associated greenhouse effect[19].

While for WPWP regions, reconstructions based on Mg/Ca and $U^{k'}_{37}$ generated similar SSTs in the EH (Supplementary Fig. 13c, d), indicating no discernible seasonality as for the modern climate, $U^{k'}_{37}$ yielded unexpected higher SST than Mg/Ca in the LG, which is also the case in other Pacific records[7,20]. It was previously argued that the warm LG temperatures interpreted from $U^{k'}_{37}$ are more likely to be proxy-specific artifacts than alkenone production shifts to warmer months[20] or shifts linked to changes in nutrient availability[7].

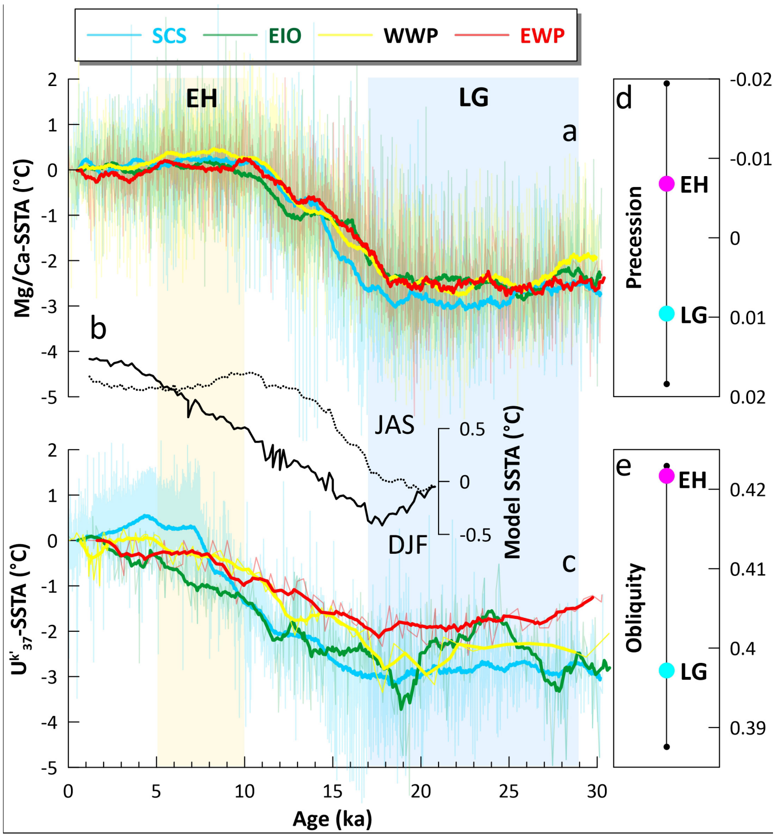

Supplementary Fig. 13. Mg/Ca and $U^{k'}_{37}$ based SSTA stacks for four regions within the IPWP during the last 30 kyr. Shadow plots in (a) and (c) show the raw data and bold SSTA stacks were computed using a sliding rectangular window of 1-kyr width by STATNARY 1.2. (b) Modeled SSTA (10°S–10°N, 105–160°E) in Jun-Aug-Sep (JAS) and Dec-Jan-Feb (DJF)[10]. (d) and (e) show mean values of precession and obliquity in the EH and LG[21].

Supplementary Tab. 2. Mean ΔSST between the EH and LG for the regions investigated. SCS, WWP, EWP and EIO represent the South China Sea, western and eastern parts of the western Pacific warm pool, as well as eastern Indian Ocean, respectively.

Sea Area	LG-EH ΔSST (°C)		
	Mg/Ca based	$U^{k'}_{37}$ based	Mixed proxies based
SCS	**-2.97** ± 0.78	**-2.38** ± 0.72	**-2.68** ± 0.81
WWP	**-2.67** ± 0.65	**-1.50** ± 0.59	**-2.59** ± 0.71
EWP	**-2.29** ± 0.67	**-0.84** ± 0.56	**-2.11** ± 0.82
EIO	**-1.92** ± 0.76	**-1.25** ± 0.58	**-1.78** ± 0.77

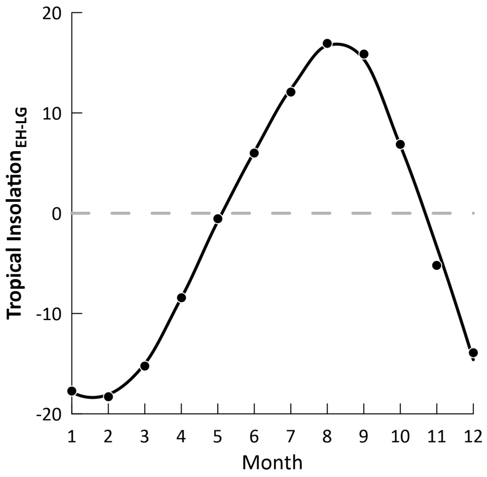

Supplementary Fig. 14. Tropical (20°N–20°S) insolation difference between the EH and LG.

3. Estimation of ocean heat content (OHC) in core KX22-4

3.1 Age model

Core KX22-4 (0.03°S, 159.24°E, water depth 2362 m) was retrieved from the central WPWP during Cruise KX08-973 of the R/V "Science-1 Vessel"[3]. We added two AMS ^{14}C age control points to the established age model[3] to better constrain the deglaciation using the planktonic foraminifera *T. sacculifer* (355–425 μm) (Table s3). Radiocarbon analysis was performed at NOSMAS and Beta Analytic Inc., USA. Radiocarbon ages were converted to calendar ages using CALIB 8.1.0 program and updated MARINE20 calibration[22], taking the local reservoir (ΔR) from the most-nearby ages of modern seawater (http://calib.org/marine) into account.

Supplementary Tab. 3. Radiocarbon dates of core KX22-4.

Depth (cm)	Lab code	Uncorrected ^{14}C age (yr B.P. ±1σ)	Local reservoir (ΔR) (yr, ±1σ)	Calendar age (yr B.P.)	Range (1σ) (yr B.P.)
**5-6***	OS-101065	4120±25	20±177	3964	3717-4210
**13-14***	OS-101066	5770±25	20±177	5961	5755-6167
**26-27***	OS-101067	9820±25	20±177	10569	10300-10838
**30-31**	Beta-566811	12610±40	20±177	14042	13755-14328
**35-36**	Beta-566812	13840±40	20±177	15849	15597-16102
**39-40***	OS-101068	17700±65	20±177	20448	20214-20682
**49-50***	OS-101081	23800±85	20±177	27160	26985-27334

*data from Zhang et al., 2017[3].

3.2 Estimation of foraminiferal apparent calcification depth

Five planktonic foraminiferal species (*G. ruber*, *Neogloboquadrina dutertrei*, *Globigerinella siphonifera*, *Globorotalia crassaformis* and *Globorotalia truncatulinoides* dextral) dwelling at different depths across the water column were selected for estimation of OHC in the upper ocean in core KX22-4. We first estimated the calcification depth of each species. For that purpose, the core-top sample of KX22-4 was used to assign the apparent calcification depth (ACD) of the above-mentioned species. We additionally used surface samples (0–2 cm) from three nearby box cores, because deep dwelling species were rare in core KX22-4. The three box cores included EQ-26 (0.00°S, 156.00°E, water depth 1945 m), EQ-28 (0.00°S, 158.00°E, water depth 2225 m) and EQ-29 (0.02°S, 159.25°E, water depth 2423 m) recovered from the Ontong-Java Plateau (OJP) during cruise NORC2019-09 of the R/V *KEXUE*.

About 30 specimens within the size fraction of 355–500 μm were prepared for stable oxygen isotope and Mg/Ca measurements. All pretreatment and analytical procedure followed the method described by Zhang et al. 2017[3]. $\delta^{18}O$ was measured by Finnigan-MAT253 mass spectrometer equipped with an automatic carbonate preparation device (Kiel Ⅲ) at the State Key Laboratory of Marine Geology, Tongji University with a standard deviation of 0.07‰. $\delta^{18}O$ results were calibrated to PDB scale by the NBS-19 standard.

Under the assumption of isotopic equilibrium, we estimated foraminiferal ACD by matching

the measured $\delta^{18}O$ values of late Holocene (<5 ka) with the predicted $\delta^{18}O_{eq}$ profile calculated using the empirical equation from Shackleton (1974)[23]:

$$SST = 16.9 - 4.38 \times (\delta^{18}O_{eq} - \delta^{18}O_{sw} + 0.27) + 0.1 \times (\delta^{18}O_{eq} - \delta^{18}O_{sw} + 0.27)^2 \quad (1).$$

$\delta^{18}O_{sw}$ as calculated by the relationship built in tropical Pacific[24]:

$$\delta^{18}O_{sw} = 0.27 \times \text{Salinity} - 8.88 \quad (2).$$

The annual mean temperature and salinity data of our site are from the SODA reanalysis dataset (v2.2.4, http://apdrc.soest.hawaii.edu/datadoc/soda_2.2.4.php). The result allocated the averaged ACDs of ~30 m water depth for *G. ruber*, ~80 m for *G. siphonifera*, ~130 m for *N. dutertrei*, ~330 m for *G. crassaformis* and ~540 m for *G. truncatulinoides*, respectively (Supplementary Fig. 15), generally consistent with previous works[4,25-27].

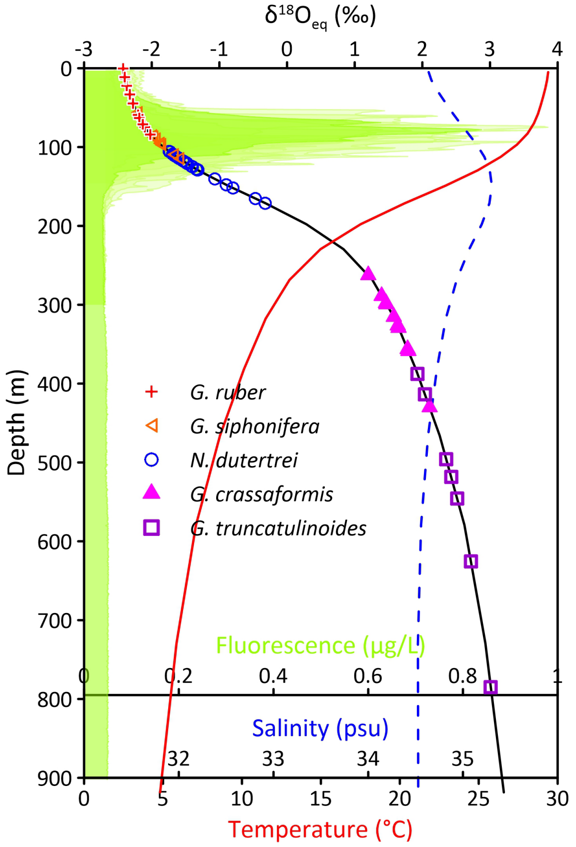

Supplementary Fig. 15. Estimation of foraminiferal calcification depth in the vicinity of core KX22-4. The temperature and salinity data are from SODA reanalysis dataset (v2.2.4, http://apdrc.soest.hawaii.edu/datadoc/soda_2.2.4.php). The chlorophyll concentration represents in situ data from 155°E–165°E, 2°S–2°N[28].

3.3 Calculation of the upper ocean heat content (OHC) in core KX22-4

The Mg/Ca ratio was measured using Inductively Coupled Plasma-Optical Emission Spectrometry (ICP-OES, Icap6300 radial; Thermo-Fisher) at the Institute of Oceanology, Chinese Academy of Science. The analytical precision of Mg/Ca ratio was 0.44% (1σ, RSD). Al/Ca, Fe/Ca and Mn/Ca were measured alongside with Mg/Ca to monitor the cleaning efficiency. None of these ratios show a correlation to Mg/Ca, indicating that the samples were not contaminated. In addition, studies from the WPWP have confirmed that the effect of salinity on Mg/Ca is negligible in this area[4,29,30]. Both the shell weight data of core KX22-4[31] and deepwater $\Delta[CO_3^{2-}]$ reconstruction at site GGC 15 (0°S, 158°E, water depth 2311 m) indicated relatively stable carbonate chemistry conditions during the last 30 kyr[32], implying that the Mg/Ca temperature are not substantially affected by carbonate dissolution.

Variations in seawater temperature at different depths since 30 ka were calculated based on Mg/Ca values of each species (Supplementary Fig. 16). The choice of foraminiferal Mg/Ca-temperature equations depends on the fitness between calculated core-top temperature and the in situ temperature at the given ACD of each species. As a consequence, equations:

$$Mg/Ca = 0.26\times\exp(0.097\times T) \text{ and } Mg/Ca = 0.21\times\exp(0.097\times T) \quad (3)$$

from Hollstein et al. (2017)[4] were used for *G. ruber* and *N. dutertrei*. Equation:

$$Mg/Ca = 0.455\times\exp(0.077\times T) \quad (4)$$

from Anand et al. (2003)[27] was used for *G. siphonifera*, and equation:

$$Mg/Ca = 0.78\times\exp(0.052\times T) \quad (5)$$

from Cléroux et al. (2013)[33] was used for *G. crassaformis*. For *G. truncatulinoides*, existing equations derived for the Atlantic[27,33-37] either underestimate or overestimate the in situ temperature in our study area. As the mean value of core-top temperatures reconstructed by equations from the tropical and north Atlantic[36,37] fits the in situ temperature well, we developed a new equation combining the two empirical equations for *G. truncatulinoides*:

$$Mg/Ca = 0.69\times\exp(0.09\times T) \quad (6)$$

with an uncertainty of ±1°C. The temperature calculated with this new equation matches the intermediate water temperature reconstructed in the WPWP at about 600 m[38].

The OHC of WPWP from core KX22-4 during the last 30 kyr (Supplementary Fig. 17) was

then calculated using the following equation:

$$OHC\ (joules) = \rho \times V \times C_p \times (T_{h1} + T_{h0}) / 2 \times (h_1 - h_0) \qquad (7)$$[39],

where ρ is the seawater density (1024 kg/m^3), V is the area of the WPWP (28.74 × 10^{12} m^2) (http://ngdc.noaa.gov/mgg/global/etopo1_ocean_volumes.html), Cp is the seawater heat content (4,000 joules/°C/kg), T_h is the water temperature at a given water depth (°C).

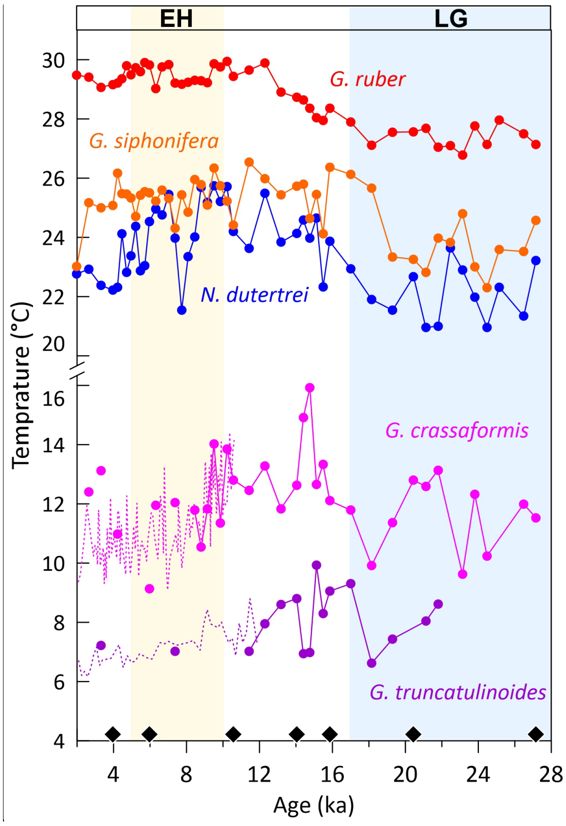

Supplementary Fig. 16. Variations in temperature at different water depths based on different foraminiferal species. The dots show the results of core KX22-4 from this study. The magenta dotted line represents intermediate water temperature based on benthic foraminiferal Mg/Ca from core CDH23 at water depth of 374 m in the EEP[40]. The purple dotted line represents intermediate water temperature based on benthic foraminiferal Mg/Ca from core 10GGC at water depth of 649 m in the WPWP[38]. Black diamonds are ^{14}C age control points.

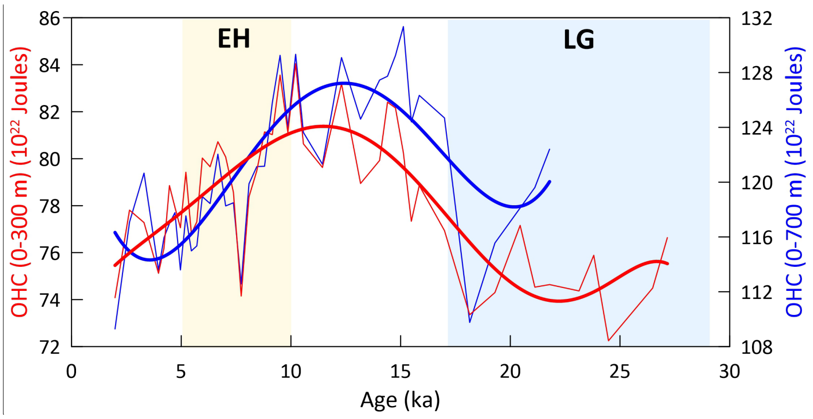

Supplementary Fig. 17. Variations in OHC in the 0–300 m and 0–700 m water column in the WPWP from core KX22-4. Bold lines are polynomial fitting results for the raw data.

REFERENCES

1 Liu, Z. *et al.* Transient simulation of last deglaciation with a new mechanism for Bølling-Allerød warming. *Science* **325**, 310-314 (2009).

2 Locarnini, R. *et al.* World ocean atlas 2018, volume 1: temperature. *NOAA Atlas NESDIS* **81**, 52 (2018).

3 Zhang, S. *et al.* Correspondence between the ENSO-like state and glacial-interglacial condition during the past 360 kyr. *Chin. J. Ocean. Limnol.* **35**, 1018-1031 (2017).

4 Hollstein, M. *et al.* Stable oxygen isotopes and Mg/Ca in planktic foraminifera from modern surface sediments of the western Pacific warm pool: implications for thermocline reconstructions. *Paleoceanography* **32**, 1174-1194 (2017).

5 Bemis, B. E., Spero, H. J., Bijma, J. & Lea, D. W. Reevaluation of the oxygen isotopic composition of planktonic foraminifera: experimental results and revised paleotemperature equations. *Paleoceanography* **13**, 150-160 (1998).

6 Spratt, R. M. & Lisiecki, L. E. A late Pleistocene sea level stack. *Clim. Past* **12**, 1079-1092 (2016).

7 Davis, C. V. *et al.* Sea surface temperature across the subarctic north Pacific and marginal seas through the past 20,000 years: a paleoceanographic synthesis. *Quat. Sci. Rev.* **246**, 106519 (2020).

8 Lee, K. E. *et al.* Roles of insolation forcing and CO_2 forcing on late Pleistocene seasonal sea surface temperatures. *Nature Communications* **12**, 5742 (2021).

9 Huang, C.-Y. *et al.* Deep sea and lake records of the Southeast Asian paleomonsoons for the last 25 thousand years. *Earth Planet. Sci. Lett.* **146**, 59-72 (1997).

10 Timmermann, A., Sachs, J. & Timm, O. E. Assessing divergent SST behavior during the last 21 ka derived from alkenones and *G. ruber*-Mg/Ca in the equatorial Pacific. *Paleoceanography* **29**, 680-696 (2014).

11 Kim, R. A., Lee, K. E. & Bae, S. W. Sea surface temperature proxies (alkenones, foraminiferal Mg/Ca, and planktonic foraminiferal assemblage) and their implications in the Okinawa Trough. *Progress in Earth and Planetary Science* **2**, 43 (2015).

12 Bova, S., Rosenthal, Y., Liu, Z., Godad, S. P. & Yan, M. Seasonal origin of the thermal maxima at the Holocene and the last interglacial. *Nature* **589**, 548-553 (2021).

13 Kawahata, H., Nishimura, A. & Gagan, M. K. Seasonal change in foraminiferal production in the western equatorial Pacific warm pool: evidence from sediment trap experiments. *Deep Sea Research Part II: Topical Studies in Oceanography* **49**, 2783-2800 (2002).

14 Mohtadi, M. *et al.* Low-latitude control on seasonal and interannual changes in planktonic foraminiferal flux and shell geochemistry off south Java: a sediment trap study. *Paleoceanography* **24**, PA1201 (2009).

15 Lin, H.-L., Wang, W.-C. & Hung, G.-W. Seasonal variation of planktonic foraminiferal isotopic composition from sediment traps in the South China Sea. *Mar. Micropaleontol.* **53**, 447-460 (2004).

16 Mohiuddin, M. M., Nishimura, A., Tanaka, Y. & Shimamoto, A. Regional and interannual productivity of biogenic components and planktonic foraminiferal fluxes in the northwestern Pacific Basin. *Mar. Micropaleontol.* **45**, 57-82 (2002).

17 Ganssen, G. M. *et al.* Quantifying sea surface temperature ranges of the Arabian Sea for the past 20,000 years. *Clim. Past* **7**, 1337-1349 (2011).

18 Hällberg, P. L., Schenk, F., Yamoah, K. A., Kuang, X. & Smittenberg, R. H. El Niño-like conditions and seasonal aridity in the Indo-Pacific warm pool during the Younger Dryas. *Clim. Past Discuss.* **2021**, 1-26 (2021).

19 Braconnot, P. *et al.* Results of PMIP2 coupled simulations of the Mid-Holocene and Last Glacial Maximum–Part 2: feedbacks with emphasis on the location of the ITCZ and mid- and high latitudes heat budget. *Clim. Past* **3**, 279-296 (2007).

20 Inagaki, M., Yamamoto, M., Igarashi, Y. & Ikehara, K. Biomarker records from core GH02-1030 off Tokachi in the northwestern Pacific over the last 23,000 years: environmental changes during the last deglaciation. *Journal of Oceanography* **65**, 847-858 (2009).

21 Laskar, J. *et al.* A long-term numerical solution for the insolation quantities of the Earth. *Astronomy & Astrophysics* **428**, 261-285 (2004).

22 Heaton, T. J. *et al.* MARINE20—The Marine radiocarbon age calibration curve (0–55,000 cal BP). *Radiocarbon*, 1-42 (2020).

23 Shackleton, N. Attainment of isotopic equilibrium between ocean water and the benthonic foraminifera genus *Uvigerina*: isotopic changes in the ocean during the last glacial. *Colloques Internationaux du C.N.R.S.* **219**, 203-209 (1974).

24 LeGrande, A. N. & Schmidt, G. A. Global gridded data set of the oxygen isotopic composition in seawater. *Geophys. Res. Lett.* **33**, L12604 (2006).

25 Rippert, N. *et al.* Constraining foraminiferal calcification depths in the western Pacific warm pool. *Mar. Micropaleontol.* **128**, 14-27 (2016).

26 Sagawa, T., Yokoyama, Y., Ikehara, M. & Kuwae, M. Shoaling of the western equatorial Pacific thermocline during the last glacial maximum inferred from multispecies temperature reconstruction of planktonic foraminifera. *Palaeogeography, Palaeoclimatology, Palaeoecology* **346–347**, 120-129 (2012).

27 Anand, P., Elderfield, H. & Conte, M. H. Calibration of Mg/Ca thermometry in planktonic foraminifera from a sediment trap time series. *Paleoceanography* **18**, 1050 (2003).

28 Bonnet, S., Biegala, I. C., Dutrieux, P., Slemons, L. O. & Capone, D. G. Nitrogen fixation in the western equatorial Pacific: rates, diazotrophic cyanobacterial size class distribution, and biogeochemical significance. *Global Biogeochemical Cycles* **23**, GB3012 (2009).

29 Zhang, P. *et al.* Geochemical characteristics from tests of four modern planktonic

foraminiferal species in the Indonesian Throughflow region and their implications. *Geoscience Frontiers* **10**, 505-516 (2019).

30 Arbuszewski, J., deMenocal, P., Kaplan, A. & Farmer, E. C. On the fidelity of shell-derived $\delta^{18}O_{seawater}$ estimates. *Earth Planet. Sci. Lett.* **300**, 185-196 (2010).

31 Qin, B., Li, T., Xiong, Z., Algeo, T. J. & Chang, F. Deepwater carbonate ion concentrations in the western tropical Pacific since 250 ka: evidence for oceanic carbon storage and global climate influence. *Paleoceanography* **32**, 351-370 (2017).

32 Yu, J. *et al.* Responses of the deep ocean carbonate system to carbon reorganization during the last glacial–interglacial cycle. *Quat. Sci. Rev.* **76**, 39-52 (2013).

33 Cléroux, C., deMenocal, P., Arbuszewski, J. & Linsley, B. Reconstructing the upper water column thermal structure in the Atlantic ocean. *Paleoceanography* **28**, 503-516 (2013).

34 Reynolds, C. E., Richey, J. N., Fehrenbacher, J. S., Rosenheim, B. E. & Spero, H. J. Environmental controls on the geochemistry of *Globorotalia truncatulinoides* in the Gulf of Mexico: implications for paleoceanographic reconstructions. *Mar. Micropaleontol.* **142**, 92-104 (2018).

35 Cléroux, C. *et al.* Mg/Ca and Sr/Ca ratios in planktonic foraminifera: proxies for upper water column temperature reconstruction. *Paleoceanography* **23** (2008).

36 Elderfield, H. & Ganssen, G. Past temperature and $\delta^{18}O$ of surface ocean waters inferred from foraminiferal Mg/Ca ratios. *nature* **405**, 442-445 (2000).

37 Regenberg, M., Steph, S., Nürnberg, D., Tiedemann, R. & Garbe-Schönberg, D. Calibrating Mg/Ca ratios of multiple planktonic foraminiferal species with $\delta^{18}O$-calcification temperatures: paleothermometry for the upper water column. *Earth Planet. Sci. Lett.* **278**, 324-336 (2009).

38 Rosenthal, Y., Linsley, B. K. & Oppo, D. W. Pacific ocean heat content during the past 10,000 years. *Science* **342**, 617-621 (2013).

39 Yang, Y., Xiang, R., Zhang, L., Zhong, F. & Zhang, M. Is the upward release of intermediate ocean heat content a possible engine for low-latitude processes? *Geology* **48**, 579-583 (2020).

40 Kalansky, J., Rosenthal, Y., Herbert, T., Bova, S. & Altabet, M. Southern Ocean contributions to the eastern equatorial Pacific heat content during the Holocene. *Earth Planet. Sci. Lett.* **424**, 158-167 (2015).
